# UGT8/GalCer-dependent resistance of breast cancer cells to drug-induced apoptosis is potentially regulated by the LIM/homeobox protein LHX6

**DOI:** 10.1038/s41598-026-42260-1

**Published:** 2026-03-04

**Authors:** Jaroslaw Suchanski, Weronika Woldanska, Safoura Nour Ebad, Krzysztof Grzymajlo, Aleksandra Piotrowska, Tomasz B. Owczarek, Hanna Romanowicz, Beata Smolarz, Piotr Dziegiel, Maciej Ugorski

**Affiliations:** 1 Department of Biochemistry and Molecular Biology, Wroclaw University of Environmental and Life Sciences, C.K. Norwida 31, 50-375 Wroclaw, Poland; 2 Division of Histology and Embryology, Department of Human Morphology and Embryology, Faculty of Medicine, Wroclaw Medical University, 50-368 Wroclaw, Poland; 3 Department of Pathology, Polish Mother Memorial Hospital-Research Institute, 93-338 Lodz, Poland; 4 Present Address: Regeneron Pharmaceuticals, 777 Old Saw Mill River Road, Tarrytown, NY 10591 USA

**Keywords:** Breast cancer, LHX6, UGT8, Transcriptional regulation, Apoptosis, Breast cancer, Tumour biomarkers

## Abstract

Ceramide galactosyltransferase (UGT8) is overexpressed in basal-like breast cancer (BC) tumors and is associated with an increased risk of lung metastasis. UGT8 synthesizes galactosylceramide (GalCer), an anti-apoptotic molecule that promotes BC cell survival within the tumor microenvironment and enhances resistance to anticancer drugs. In this study, we aimed to elucidate the molecular mechanisms underlying UGT8 overexpression in BC cells. *UGT8* promoter constructs and deletion mutants were generated by PCR. Promoter activity was assessed using a dual-luciferase assay, and key regulatory sequences were identified by electrophoretic mobility shift assay (EMSA). Candidate transcription factors were predicted using the JASPAR database and validated by qPCR, Western blotting, and immunohistochemistry. GalCer levels were measured by thin-layer chromatography (TLC) binding assay, and LHX6–DNA interactions were analyzed by surface plasmon resonance. Apoptotic cells were detected using the Thermo Dead Cell Apoptosis Kit. *UGT8* promoter activity was significantly higher in UGT8-positive MDA-MB-231 cells than in UGT8-negative T47D and MCF-7 cells, indicating that *UGT8* gene expression is regulated at the transcriptional level. Sequential promoter deletions combined with EMSA localized a key regulatory region (− 1132 to − 1618 bp), termed the UGT8 response element (UGT8RE). In silico analysis identified potential transcription factors, among which the LIM/homeobox protein LHX6 was markedly upregulated in MDA-MB-231 cells. Site-directed mutagenesis of two predicted LHX6 binding sites (LHX6BS1 and LHX6BS2) demonstrated that LHX6BS2 is essential for UGT8 promoter activity. RNAi-mediated inhibition of LHX6 reduced UGT8 expression and GalCer synthesis, thereby sensitizing MDA-MB-231 cells to doxorubicin-induced apoptosis. The LIM/homeobox protein LHX6 may regulate the UGT8 expression in BC cells. Targeting LHX6 decreases UGT8 expression and GalCer synthesis, thereby sensitizing MDA-MB-231 cells to doxorubicin. Given UGT8’s role in cell survival and drug resistance, inhibition of LHX6 may represent a promising therapeutic strategy for drug-resistant BC.

**Supplementary Information:**

The online version contains supplementary material available at 10.1038/s41598-026-42260-1.

## Introduction

Ceramide galactosyltransferase (UGT8, E.C. 2.4.1.45) is the enzyme responsible for the synthesis of galactosylceramide (GalCer)^1,2^, which is best known as a major component of myelin and plays an essential role in stabilizing and maintaining its proper structure^3–5^. Consistent with these findings, high UGT8 expression has been demonstrated in oligodendrocytes and Schwann cells^6^. However, aberrant UGT8 expression has also been reported in several types of cancer^7–10^, including breast cancer (BC). In BC, UGT8 overexpression has been associated with estrogen receptor α (ER)-negative status^11,12^, and transcriptional profiling of triple-negative breast cancer (TNBC) has shown elevated UGT8 expression^13–15^. Furthermore, studies on organ-specific metastatic patterns in BC demonstrated that UGT8 expression is markedly increased in a subset of tumors representing the basal-like subtype and is part of a six-gene signature predicting BC lung metastasis^7,16,17^.

Importantly, there is evidence that GalCer synthesized by UGT8 is directly involved in breast cancer progression^18^. BC cells with high GalCer levels were shown to form primary tumors and metastatic colonies more efficiently than cells with absent or low expression of this glycosphingolipid (GSL), which was associated with reduced apoptosis in the former. This and subsequent studies^19^ demonstrated that GalCer functions as an anti-apoptotic molecule, suggesting that its presence promotes cell survival within a hostile tumor microenvironment. Moreover, GalCer increases the resistance of BC cells to anticancer drugs, including doxorubicin, paclitaxel, and cisplatin (cis-diamminedichloroplatinum), indicating that GalCer may contribute to the drug resistance of UGT8-overexpressing BC cells^20^.

Several lines of evidence suggest that *UGT8* gene expression is regulated at the transcriptional level^21–25^. Using human oligodendroglioma HOG cells, which express UGT8, and human neuroblastoma LAN-5 cells, which do not express this enzyme, the *UGT8* gene promoter was found to function in a cell-specific manner^22^. GC-box and CRE motifs, acting in concert, were the essential elements responsible for UGT8 expression in oligodendroglioma cells^23^. A more recent study revealed that UGT8 expression in this cell type is regulated explicitly by two transcription factors (TFs): Nkx2.2, which acts as a positive regulator, and OLIG2, which acts as a negative regulator^24^. The regulatory mechanisms responsible for the expression of UGT8 were also investigated in non-small lung cancer (NSCLC)^25^. It was found that the key TF responsible for the up-regulation of *UGT8* gene expression in NSCLC cells turned out to be SOX-9. All these data underline that UGT8 expression and, therefore, GalCer biosynthesis are regulated in a cell-type and stage-specific manner. The regulatory mechanism responsible for the overexpression of the *UGT8* gene and thus the accumulation of GalCer in BC cells is still unknown. Since BC tumors with elevated GalCer levels are resistant to chemotherapy^20^, understanding the molecular mechanisms responsible for the increased expression of UGT8 in a subset of BC tumors with organ-specific metastasis to the lung^8–10^ is important not only for a better understanding of BC biology but also from a clinical point of view. Therefore, the present studies were undertaken to identify key molecular mechanisms responsible for the overexpression of UGT8 in BC cells.

## Materials and methods

### Cell lines and culture conditions

The BC MDA-MB-231 and T47D cell lines were obtained from the American Type Culture Collection (ATCC). The BC MCF7 cell line was obtained from the Cell Line Collection of the Hirszfeld Institute of Immunology and Experimental Therapy (Wroclaw, Poland). MCF7 cells were authenticated by the ATCC Cell Line Authentication Service using short tandem repeat analysis. MDA-MB-231 and MCF7 cells were cultured in α-MEM supplemented with 10% fetal bovine serum (FBS, Cat. No. S181H, Biowest), 2 mM L-glutamine, and antibiotics. T47D cells were cultured in RPMI-1640 medium supplemented with 10% FBS, 2 mM L-glutamine, bovine insulin (0.2 U/mL) (Cat. No. I6634, Merck) and antibiotics.

### Breast cancer tissue specimens

The present study was conducted on 339 breast cancer tissues embedded in paraffin blocks. Material was obtained from patients diagnosed and treated at the Polish Mother’s Memorial Health Institute in Lodz in the years 2004–2012. Tumor histologic type and malignancy staging system (TNM system) were determined according to the World Health Organization (WHO) criteria^26^.

### Cloning of UGT8 promoter region and construction of promoter/luciferase reporter vectors

The promoter region of the *UGT8* gene^22^ was cloned by amplification of the genomic sequence by PCR using total DNA purified from MDA-MB-231 cells as a template and forMluI-prUGT8 and revNheI-prUGT8 primers (Table S1) as follows: 35 cycles of denaturation (95 °C for 20 s), annealing (55 °C for 20 s) and extension (72 °C for 1 min). Deletion mutants of the *UGT8* promoter were obtained by PCR amplification using the *UGT8* promoter region as a template and the primers listed in Table S1 as follows: 35 cycles of denaturation (95 °C for 20 s), annealing (55 °C for 20 s) and extension (72 °C for 1 kb/ min). All PCR primers contained additional sequences corresponding to an Mlu I restriction site in the 5’ primers and a Nhe I restriction site in the 3’ primers. The generated *UGT8* promoter and deletion mutants were cloned into the corresponding sites of the pGL3 Basic Vector (Cat. No. E1751, Promega).

### Reporter assay

Promoter activity was determined using the Promega Dual-Luciferase Reporter Assay System, following the protocol previously described by Suchanski et al.^27^. In brief, cells were cultured to approximately 70% confluence in 6-well plates and co-transfected with 2 µg of a pGL3 Firefly luciferase reporter plasmid and 2 µg of the pRL-TK *Renilla* luciferase control vector using polyethylenimine (PEI). After 48 h, cells were lysed in 200 µL of passive lysis buffer, and luciferase activities were measured sequentially using a Tecan luminescence microplate reader with a 1 s integration time per well. Mock-transfected cells and empty wells served as background controls. Firefly luciferase activity was normalized to *Renilla* luciferase activity. All experiments were performed in three independent biological replicates.

### Nuclear extracts and electrophoretic mobility shift assay (EMSA)

Nuclear and cytoplasmic fractions were isolated using the NE-PER Nuclear and Cytoplasmic Extraction Kit (Cat. No.78833, Thermo) according to the protocol previously delineated by Suchanski et al.^27^. Fraction purity was verified using GAPDH and histone H3 as cytoplasmic and nuclear markers, respectively. Biotin-labeled double-stranded oligonucleotides were generated by PCR with 5′-biotinylated primers (Table S1) and purified prior to use. Binding reactions containing 5 µg nuclear extract, 1 µg poly(dI–dC), and 2 nM biotin-labeled DNA were performed in Tris-based binding buffer (pH 7.5) supplemented with KCl, MgCl₂, dithiothreitol, Nonidet P-40, and glycerol, and incubated at 23 °C for 20 min. DNA–protein complexes were resolved on a 6% Tris–borate–EDTA (TBE) polyacrylamide gel (100 V, 1 h), transferred to a nylon membrane, and detected using the LightShift chemiluminescent EMSA kit (Cat. No. 20148, Thermo).

### Quantitative PCR (qPCR) assay

The purification of RNA from BC cells was performed using the RNeasy Mini Kit (Cat. No. 74104, Qiagen), following the manufacturer’s instructions. To synthesize cDNA, the SuperScript RT (Thermo Fisher Scientific) was utilized. The relative amounts of mRNAs were determined by qPCR with the EvaGreen dye-based detection system (Cat. no. 31000, Biotium) according to the manufacturer’s protocol, using an iQ5 Optical System (Biorad). GAPDH was used to normalize RNA levels, and the primers are listed in Table S1. The PCR amplification protocol involved an initial denaturation step at 95 °C for three minutes, followed by 35 cycles of 20 s at 95 °C, 20 s at 55 °C, and 20 s at 72 °C. The relative changes in gene expression were subsequently calculated using the ΔΔCt (cycle threshold) method. The calculation of fold change values was performed using the 2-ΔΔCt formula.

### Western blotting

The Western blotting was executed by the protocol previously delineated by Suchanski et al.^19^. In summary, cells were lysed in 100 μL of RIPA lysis buffer (Cat. no. R0278, Merck) to yield soluble proteins. The bicinchoninic acid assay (Merck) was used to quantify these soluble proteins. Subsequently, cell lysates (UGT8) and nuclear extracts (GSX1, SOX4 and LHX6) were subjected to SDS-PAGE (12%), and the separated proteins were transferred to 0.45 µm nitrocellulose membranes (Cytiva). To detect specific proteins, the membrane slices were incubated with appropriate primary antibodies: anti-UGT8 (Cat. No. orb214717, Biorbyt), anti-GSX1 (Cat. No. BS-11612R, Thermo), anti-SOX4 (Cat. No. PA595290, Thermo), anti-LHX6 (Cat. No. orb1535192, Biorbyt) and mouse monoclonal anti-GAPDH antibody (Cat. No. NB300-221, Novus Biologicals). Then, they were incubated with HRP-conjugated goat polyclonal antibodies directed against rabbit (Cat. No. P0448, Dako) or murine (Cat. No. 115–035-003, Jackson ImmunoResearch) immunoglobulins at room temperature for 1 h. The quantifications of proteins were based on GAPDH expression.

### Oligonucleotide annealing

Complementary oligonucleotides (**Table S1**) were annealed to create 50 bp double-stranded DNA fragments (ΔUGT8RE1-50 or UGT8RE1-50). Initially, equimolar concentrations of complementary oligonucleotides (100 µM each) were mixed at a 1:1 molar ratio in a sterile microcentrifuge tube. The resulting oligonucleotide mixture was then diluted to a final concentration of 2 µM using buffer consisting 10 mM Tris, 1 mM EDTA, 50 mM NaCl (pH 8.0). The annealing process was conducted as follows: 5 min at 95 °C, followed by 72 cycles of 1 min at 95 °C with the temperature ramping down by 1 °C per cycle, and concluding by holding the temperature at 10 °C. After annealing, the double-stranded DNA probes were either aliquoted and stored at -20 °C for long-term preservation or used immediately for Surface Plasmon Resonance (SPR) assays.

### Surface plasmon resonance (SPR)

The binding of a recombinant protein with an N-terminal GST tag corresponding to the amino acid sequence 274–363 of human LHX6 (Cat. No. H00026468-Q01-25ug, Novus Biologicals) to double-stranded DNA fragments (ΔUGT8RE1-50 or UGT8RE1-50) was analyzed using SPR with a BIAcore T200 (Cytiva).

The CM5 sensor chip surface was activated according to the manufacturer’s protocol. Shortly, the coupling solution (40 μl NHS/EDC) was injected, followed by an injection of the anti-GST antibody (αGST-Ab) and finally by an injection of ethanolamine to deactivate esters on the sensor chip surface. A solution of LHX6 fragment (10 µg/ml) was injected for 4 min at a flow rate of 10 µl/min and immobilized on the prepared surface via GST-tag to a final level of 1000 RU. To determine the affinity of the analyzed DNA fragments to LHX6, single-cycle kinetics was employed for this experiment, where increasing concentrations of analyzed DNA fragments ranging from 5 to 625 nM (association phase, 10 µl/min, 5 min per concentration) were injected without fully dissociating the previous concentration. The same samples were passed over a control chip surface with the anti-GST antibody immobilized.

All binding experiments were conducted at 25 °C with a flow rate of 10 µl/min in HBS-N running buffer supplemented with 0.05% P20 detergent at pH 4 (Cat. No. BR100054, Cytiva). The resulting sensorgrams were obtained by first subtracting the buffer blank from the curves recorded for the interactions, followed by the subtraction of DNA fragment binding to the control surface. Steady-state affinity values were determined using BIAevaluation 3.1 software.

### shRNA gene silencing

To silence the *LHX6* gene, cells were transduced with a commercially available shRNA pLKO.1.0-puro vector (Merck) containing the sequences that targeted the LHX6 gene (NM_014368), designated as MDA.shLHX6. Control cells were transduced with a non-mammalian shRNA control vector (Merck). The procedure for producing lentiviruses was the same as previously described^19^. To select for LHX6-silenced cells and control cells, puromycin (1 μg/ml) was used (Cat. no. A1113803, Merck).

### Isolation of neutral GSLs and HP-TLC-binding assay

Neutral GSLS were purified as previously described^28^ and analyzed by high-performance thin-layer chromatography (HP-TLC) on silica gel 60 HP-TLC plates (Merck). The separation of neutral GSLs was achieved through the utilization of a solvent system composed of 2-isopropanol, methyl acetate, 15 M ammonium hydroxide, and water, in a volumetric ratio of 75:10:5:15 (v/v/v/v). Visualization of GSLs was achieved by spraying the plates with a primuline reagent solution (0.05% primuline in a 4:1 acetone/water mixture, v/v), followed by heating at 120 °C for one minute. GalCer was detected via a TLC-binding assay, as previously described^18^, employing rabbit polyclonal anti-GalCer antibodies (Cat. No. MAB342, Merck).

### Apoptotic assay

A total of 5 × 10^5^ cells were plated in 96-well plates (Nunc) and allowed to adhere overnight. Thereafter, the cells were subjected to a 48-h treatment with 1 µM doxorubicin. The proportions of apoptotic cells were then quantified using the Dead Cell Apoptosis Kit (Cat. No. V35113, Thermo). Fluorescence signals were acquired on a BD FACS Lyric flow cytometer (Becton–Dickinson), and data analysis was performed using BD FACSuite™ software (Becton–Dickinson). The early apoptotic cells were identified by staining with Annexin V APC, while the late apoptotic cells were characterized by dual staining with Annexin V APC and SYTOX Green.

### Tissue microarrays (TMAs) and immunohistochemical reactions (IHC)

All IHC reactions were performed on archival paraffin blocks consisting of 339 cases of breast cancer. Initially, hematoxylin and eosin-stained slides from paraffin blocks were scanned using a Pannoramic MIDI histological scanner (3DHistech), creating virtual slides. The histopathologist selected representative spots for the microarray (3 spots from each block, each with a diameter of 1.5 mm). Tissue microarrays were performed using the automated TMA Grand Master system (3DHistech). The TMAs paraffin blocks were cut into 4 μm sections. The IHC reactions were performed using Autostainer Link 48 (Agilent). Deparaffinization, rehydration, and antigen retrieval was performed using EnVision FLEX Target Retrieval Solution (97 °C, 20 min; pH 9) in PT-Link platform (Agilent). First, to block endogenous peroxidase activity, slides were incubated 5 min. with EnVision FLEX Peroxidase-Blocking Reagent (Agilent). Polyclonal rabbit anti-LHX-6 antibody (1:400, orb1535192, Biorbyt) and polyclonal rabbit anti-UGT8 (1:1000, 17982-1-AP, Proteintech) were used as the primary antibody (20 min incubation). In the next step, the slides were incubated with EnVision FLEX/HRP (20 min). 3,3’-diaminobenzidine was used to visualize the reaction (10 min. incubation). Finally, the sections were counterstained with EnVision FLEX Hematoxylin (Agilent), dehydrated in graded ethanol alcohol (70, 96 and 99.8%) and xylene, and mounted in the Dako mounting medium (Agilent). The primary antibodies were diluted in FLEX Antibody Diluent (Agilent).

### Evaluation of IHC reactions

IHC reaction for the cytoplasmic UGT8 antigen was assessed using the immunoreactive score (IRS) scale by Remmele and Stegner^29^. The final result is multiplication of reaction intensity (0 points—no reaction; 1 point—weak reaction; 2 points—moderate intensity; 3 points—intense reaction) and the percentage of positive cells (0 points—no positive cells, 1 point – 10% of positive cells; 2 points – 11–50%; 3 points – 51–80%; 4 points – 80% of positive cells).

The nuclear expression of LHX6 was determined using a scale that analyses the percentage of the number of cancer cells with positive nuclear expression according to the following scale: (0—no expression,1 point— > 0–10%, 2 points— > 10–25%, 3 points— > 25–50%, 4 points— > 50%)^30^.

### Analysis of publicly available databases

The breast cancer cohort data from The Cancer Genome Atlas (TCGA) was accessed through cBioPortal for Cancer Genomics (https://www.cbioportal.org). Z-scored mRNA expression data for the genes LHX6 and UGT8 were downloaded, along with clinical annotations. The clinical data included molecular subtype classifications (Basal-like, HER2-enriched, Luminal A, Luminal B, and Normal-like) as defined by the TCGA consortium. Z-scores represent standardized gene expression values, calculated relative to the distribution of expression levels within the dataset, enabling cross-sample comparisons. Molecular subtype classification was utilized to stratify the cohort and analyze gene expression patterns in relation to clinical outcomes.

Survival analysis was conducted using the KMplotter multi-study cohort (https://kmplot.com), which integrates patient data from the METABRIC, TCGA, and IMPACT datasets. The cohort includes molecular subtype classifications based on the PAM50 signature. Univariate Kaplan–Meier survival analysis was performed using median gene expression levels as the cutoff to stratify patients into high- and low-expression groups. Survival outcomes, including overall survival (OS) and recurrence-free survival (RFS), were evaluated in relation to the expression levels of the genes of interest, as described in^31^.

### Statistical analysis

Statistical analysis was performed using Statistica 13 (StatSoft, Krakow, Poland) and Prism 5.0 (GraphPad Software, San Diego, CA, USA) using Kruskal–Wallis and Mann–Whitney tests. The Mann–Whitney test was employed to compare the groups of data that did not meet the assumptions of the parametric test. Statistically significant results were defined as those with a *p*-value less than 0.05. The correlations between the scores of the UGT8 and LHX6 were tested using the Spearman rank correlation test.

## Results

### Identification of the cis-elements responsible for the regulation of UGT8 expression

**Fig. 1:**
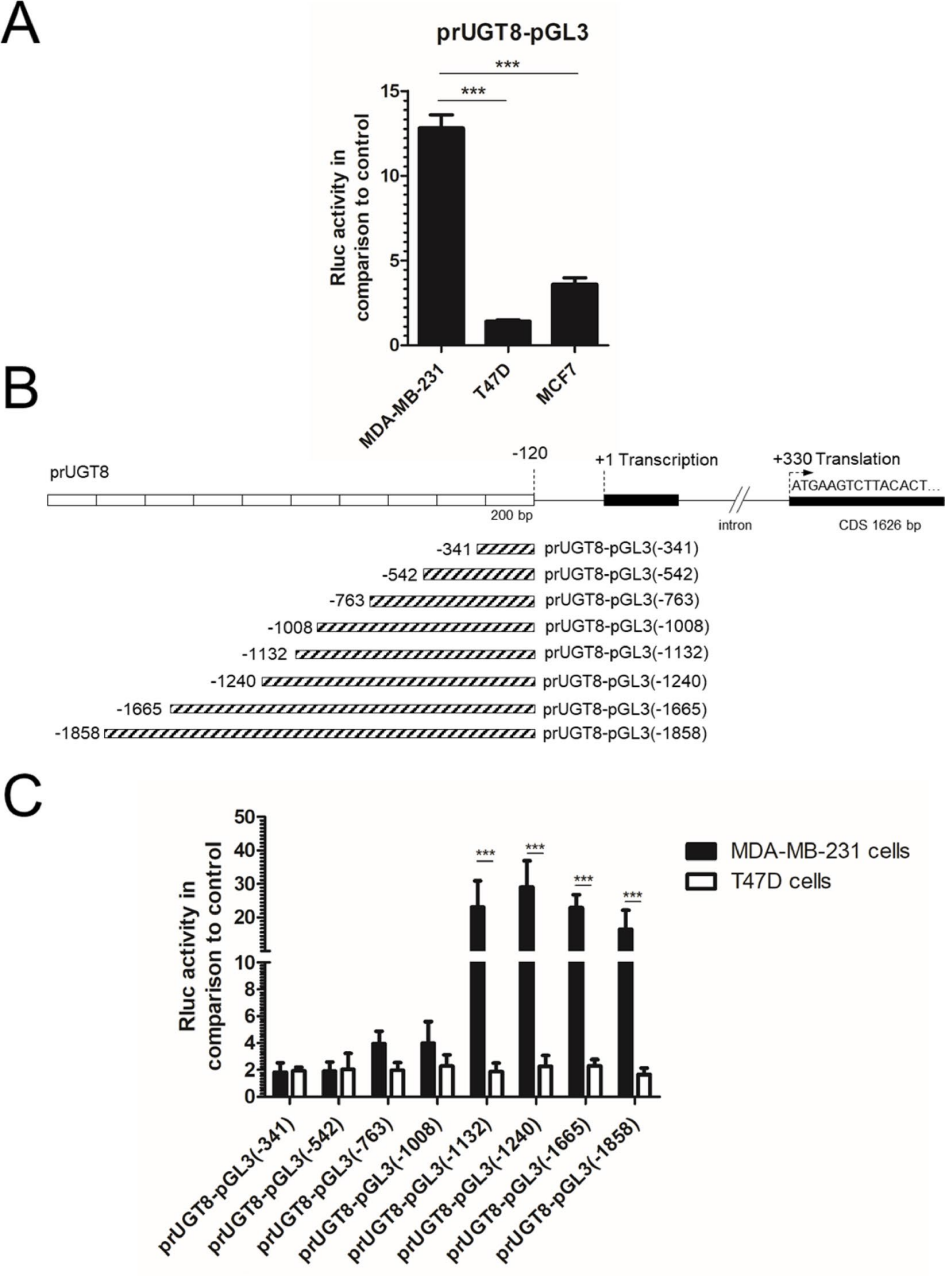
(**A**) Transcriptional activity of the prUGT8 fragment, representing the 2052 bp promoter of the *UGT8* gene, cloned into the pGL3 Basic Vector (prUGT8-pGL3) after transfection into the BC cell lines MDA-MB-231, T47D and MCF7. Promoter activities were measured using the Dual-Luciferase Reporter Assay System (Promega). The bars represent the luciferase activities compared to the control pRL-TK vector. All values are mean ± SD of at least two independent transfection experiments, each assayed in five technical replicates, ***p < 0.001. (**B**) Schematic representation of prUGT8 deletion mutants. (**C**) Luciferase activities of prUGT8 deletion mutants cloned into the pGL3 Basic Vector after transfection into MDA-MB-231 and T47D BC cell lines. The bars represent the luciferase activities compared to the control pRL-TK vector. All values are mean ± SD of at least two independent transfection experiments, each assayed in five technical replicates, ***p < 0.001.

To prove that the expression of UGT8 in BC cells is regulated at the transcriptional level^22,23^, the promoter region of the *UGT8* gene 2052 bp in length, designated as prUGT8, was cloned into the pGL3 basic vector (prUGT8-pGL3) and its activity was examined with three BC cell lines, MDA-MB-231 cells expressing UGT8 and T47D and MCF-7 cells not expressing UGT8. As shown in Fig. 1A, the activity of the prUGT8-pGL3 reported plasmid transfected into MDA-MB-231 cells was 5.8-fold and 4.4-fold higher than that of the prUGT8-pGL3 reported plasmid transfected into T47D and MCF-7 cells, respectively. To identify the *cis*-acting regulatory elements responsible for these differences, a series of sequentially deleted promoter fragments was obtained by PCR (Fig. 1B). In all DNA fragments with different 5’ ends, their 3’ ends terminated 120 nucleotides upstream of the transcription start site (+ 1). After construction of the deletion vectors, their reporter activities were examined after transfection into MDA-MB-231 and T47D cells. It was found that strong enhancer elements are present in the region between -1132 to -1665 bp from the transcription start site, as the luciferase activity of three deletion mutants: prUGT8-pGL3(-1132), prUGT8-pGL3(-1240) and prUGT8-pGL3(-1665) was highly increased compared to others (Fig. 1C). On the other hand, the lowest luciferase activity was observed for prUGT8-pGL3(-341) and prUGT8-pGL3(-542) deletion mutants, and this level of activity was termed “basal”. In contrast to MDA-MB-231 cells, transient transfection of T47D cells with the same DNA constructs showed essentially only “basal” activity for all deletion mutants analyzed.

### Characterization of TFs responsible for overexpression of UGT8 in breast cancer cells

**Fig. 2:**
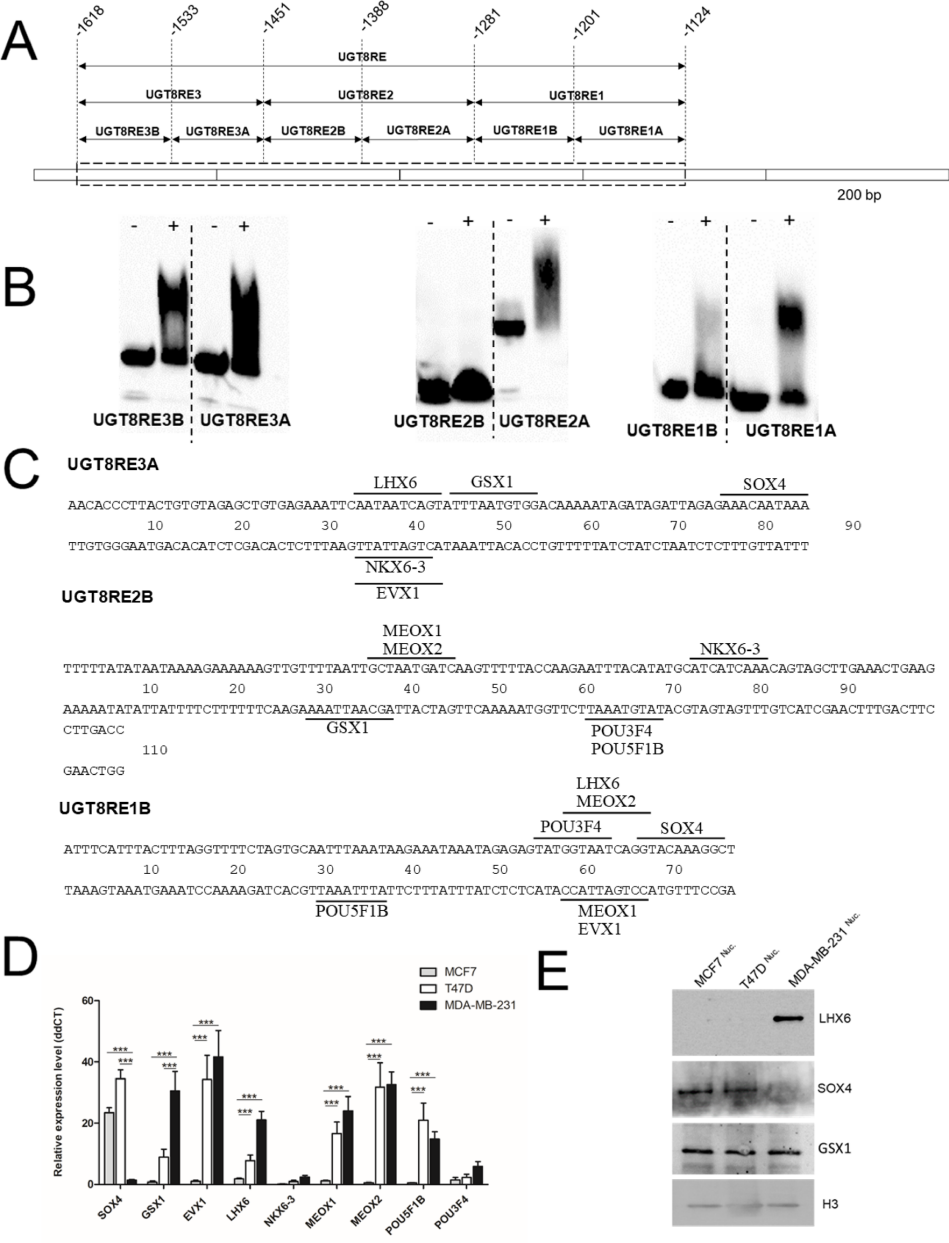
(**A**) Schematic representation of the UGT8 gene promoter region designated as the UGT8 response element (UGT8RE) as described in the text. (**B**) Binding of nuclear proteins from MDA-MB-231 cells to UGT8 response element (UGT8RE) fragments analyzed by EMSA in the presence (+) and absence (−) of nuclear lysate. EMSA was performed with double-stranded biotin-labeled oligonucleotide probes. (**C**) Transcription factors (TFs) that bind at least twice to UGT8RE. The UGT8RE3A, UGT8RE2B, and UGT8RE1B fragments were subjected to in silico analysis using the JASPAR database. (**D**) Expression of TF mRNAs in BC cell lines MCF7, T47D, and MDA-MB-231. qPCR was used to analyze TF mRNAs. Their expression levels were normalized against GAPDH, and cells with the lowest expression levels of TF were used as a calibrator sample. All values are mean ± SD of at least 2 independent experiments, each assayed in triplicate. ***p < 0.001. (**E**) Western blotting analysis of TFs expression in BC cell lines. Rabbit polyclonal antibodies anti-GSX1, anti-SOX4, and anti-LHX6 were used to detect TFs in nuclear extracts. For Western blotting, 40 μg of proteins were separated by SDS-PAGE under reducing conditions on a 12% gel and electrophoretically transferred to a nitrocellulose membrane. H3 was used as an internal control.

Given the results obtained with the prUGT8 deletion mutants, we used EMSA to determine whether the promoter region located between − 1124 and − 1618 bp from the transcription start site, termed the UGT8 response element (UGT8RE) (Fig. 2A), binds nuclear proteins from MDA-MB-231 and T47D cells. Therefore, the UGT8RE was divided into three DNA fragments corresponding to nucleotides: − 1451 to − 1618 (UGT8RE1), -1281 to -1450 (UGT8RE2), and − 1124 to − 1280 (UGT8RE3). These double-stranded DNA probes, synthesized by PCR, were incubated with nuclear extracts from MDA-MB-231 and T47D cells. It was found that nuclear proteins from MDA-MB-231 cells, in contrast to T47D cells, formed one major complex with all three UGT8REs (Fig. S1). Considering the large number of TFs that can potentially bind to the analyzed promoter sequences, they were subsequently split into two smaller fragments designated as A and B (Fig. 2A), which were subjected to further EMSA experiments. It was found that the following four fragments: UGT8RE1B (-1618/-1533), UGT8RE1A (-1532/-1451), UGT8RE2A (-1387/-1281), and UGT8RE3A (-1200/-1124) were bound by nuclear extracts of MDA-MB-231 cells (Fig. 2B). To identify key TFs that bind to the *UGT8* gene promoter, DNA fragments: UGT8RE3A, UGT8RE2A, UGT8RE1A and UGT8RE1B were subjected to in silico analysis using the JASPAR database. To limit the number of TFs that could potentially interact with the *UGT8* promoter sequence, only those with at least two binding sites in UGT8REs were further investigated (Fig. 2C). Therefore, the expression of the following TFs was examined: SOX4, LHX6, GSX1, NKX6-3, EVX1, MEOX1, MEOX2, POU5F1B, POU3F4, NKX6-3 by real-time PCR in BC MDA-MB-231 cells with high expression of UGT8 and T47D and MCF7 cells with low expression of this enzyme. Statistically significant upregulation of only two genes, GSX1 and LHX6, was observed in MDA-MB-231 cells compared to both MCF7 and T47D cells. (Fig. 2D). On the other hand, SOX4 mRNA was highly decreased in MDA-MB-231 cells compared to MCF7 and T47D cells.

Based on these results, the expression of GSX1, LHX6, and SOX4 in nuclear extracts was analyzed using Western blotting. Only LHX6 levels were significantly higher, and SOX4 levels were significantly lower in MDA-MB-231 cells compared to MCF7 and T47D cells (Fig. 2E). The notable reduction of SOX4 expression in MDA-MB-231 cells is important. However, considering the EMSA results indicating the binding of nuclear extracts from MDA-MB-231 cells to UGT8RE, and thus the presence, not the absence, of specific TFs in the nuclear lysate, only LHX6 was selected for further study.

### LHX6 increases the resistance of BC cells to drug-induced apoptosis by up-regulating the *UGT8* gene

**Fig. 3:**
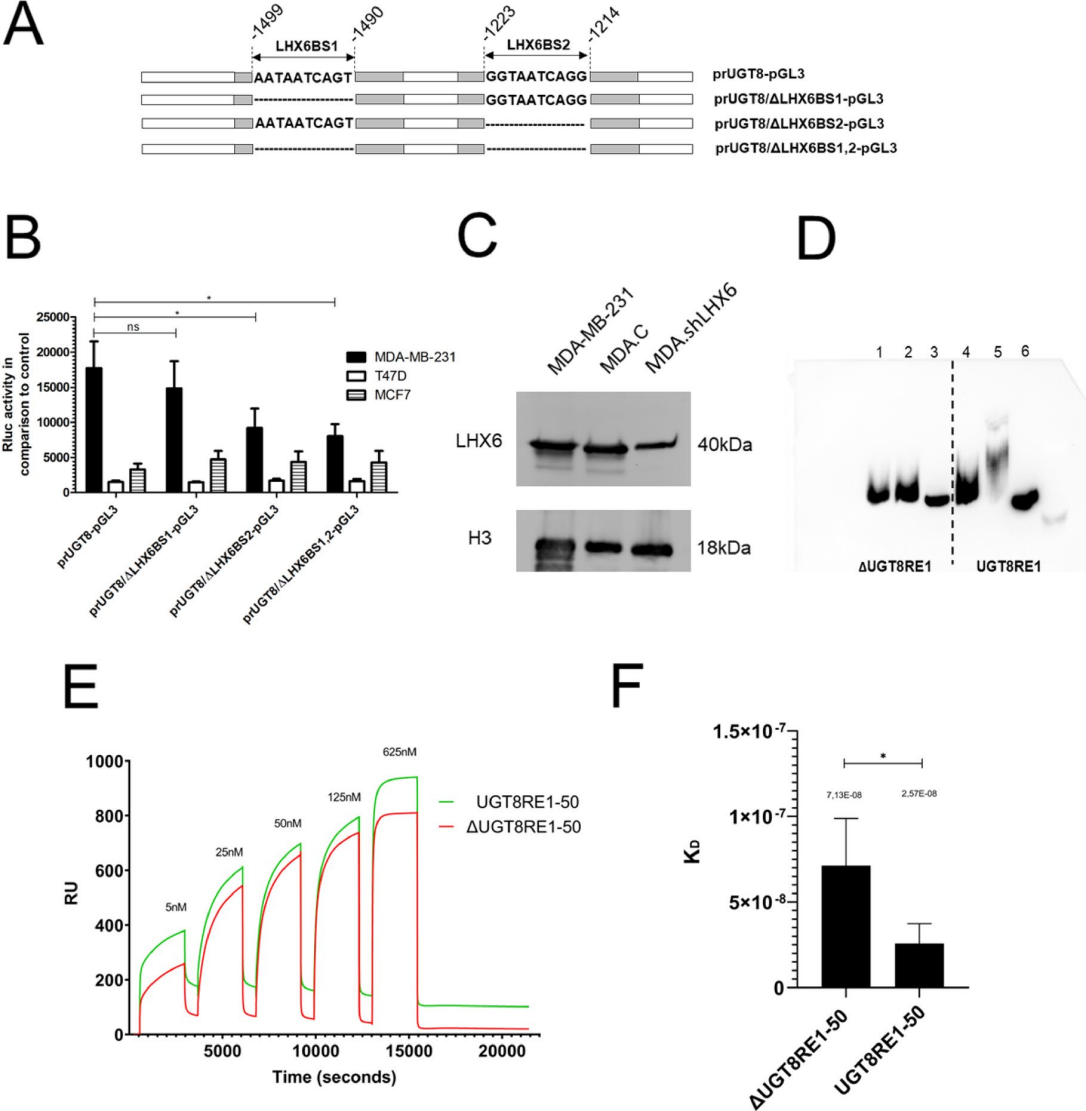
(**A**) Mutational analysis of LHX6 binding sites in the *UGT8* gene promoter. A schematic representation of the *UGT8* promoter (prUGT8) shows functional and deleted LHX6 binding sites. The binding sites for LHX6, labeled LHX6BS1 and LHX6BS2, are indicated by the sequence of the sense strand of the DNA. The sequences of the deleted binding sites are represented by dotted lines. (**B**) Luciferase activities of these deletion mutants cloned into the pGL3 Basic Vector, following transfection into the MDA-MB-231, T47D, and MCF7 BC cell lines were measured. Promoter activities were assessed using the Dual-Luciferase Reporter Assay System (Promega). The bars represent the average luciferase activities compared to the control pRL-TK vector. All values are expressed as mean ± SD from at least two independent experiments, each assayed in five technical replicates; *p < 0.05. (**C**) Western blotting analysis of LHX6 expression in wild-type MDA-MB-231 cells, MDA-MB-231 cells transfected with scrambled shRNA (MDA.C), and MDA-MB-231 cells transfected with shRNA targeting LHX6 mRNA (MDA.shLHX6). For Western blotting, 40 μg of proteins were separated by SDS-PAGE under reducing conditions on a 12% gel and then electrophoretically transferred to a nitrocellulose membrane. H3 served as an internal control. (**D**) Binding of nuclear proteins from MDA.shLHX6 cells (lanes 1 and 4) and MDA.C cells (lanes 2 and 5) to mutated UGT8RE3, which lacks the LHX6 binding site (UGT8RE), and non-mutated UGT8RE3, as analyzed by EMSA. Lanes 3 and 6 show the absence of nuclear lysates. EMSA was performed using double-stranded biotin-labeled oligonucleotide probes. (**E**) Surface plasmon resonance characteristics for the interaction between LHX6 (a recombinant protein with an N-terminal GST tag corresponding to the amino acid sequence 274–363 of human LHX6) and oligonucleotide UGT8RE1-50, which contains the LHX6 binding site, compared to oligonucleotide ΔUGT8RE1-50, which does not have the LHX6 binding site. Representative single-cycle kinetics (SCK) sensorgrams display five sequential injections of UGT8RE1-50 (green line) and ΔUGT8RE1-50 (red line) oligonucleotides. (**F**) Steady-state dissociation constant calculated from three independent experiments, *p < 0.1.

Based *on silico* analysis, the LHX6 interacts with sequences located at positions (-1499/-1490) and (-1223/-1214) of the prUGT8, and these binding sites were designated LHX6BS1 and LHX6BS2, respectively. Therefore, to prove that LHX6 is involved in the regulation of the *UGT8* gene, prUGT8 mutants with deletion of binding sites for LHX6 were obtained by site-directed mutagenesis (Fig. 3A). The following mutants were constructed: prUGT8 without LHX6BS1, designated as prUGT8/ΔLHX6BS1, prUGT8 without LHX6BS2, designated as prUGT8/ΔLHX6BS2, and prUGT8 without both binding sites, designated as prUGT8/ΔLHX6BS1,2. After cloning into the pGL3 Basic vector, the reporter plasmids designated prUGT8/ΔLHX6BS1-pGL3, prUGT8/ΔLHX6BS2-pGL3, and prUGT8/ΔLHX6BS1,2-pGL3, respectively, were analyzed for promoter activity after transfection into MDA-MB-231 cells. It was found that deletion of LHX6BS2 strongly decreased the activity of prUGT8, and this effect was not observed in the case of LHX6BS1 deletion (Fig. 3B). Consistent with this finding, the promoter activity of the prUGT8/ΔLHX6BS1,2 double mutant was comparable to that of prUGT8/ΔLHX6BS2. To demonstrate that LHX6 is the major protein species in the nuclear extract of BC cells that binds to prUGT8, MDA-MB-231 cells with inhibited expression of the *LHX6* gene were obtained with shRNA and named MDA.shLHX6 (Fig. 3C). Although shRNA-mediated knockdown did not completely silence the *LHX6* gene, the reduction in protein levels was sufficient to prevent detectable binding to the UGT8RE3 fragment of prUGT8 in the EMSA assay, whereas such binding was observed in the case of untreated cells (Fig. 3D).

To further demonstrate that LHX6 binds to the *UGT8* promoter, a recombinant protein featuring an N-terminal GST tag corresponding to the amino acid sequence 274–363 of human LHX6, along with the 50 bp ΔUGT8RE1 and UGT8RE1 fragments, referred to as ΔUGT8RE1-50 and UGT8RE1-50 respectively (see supplementary materials), underwent SPR analysis. Single-cycle sensorgrams were captured by injecting increasing concentrations of oligonucleotides over the immobilized LHX6 fragment as described in Materials and Methods. The representative sensorgram is displayed in the figure (Fig. 3E). As expected, the UGT8RE1-50 oligonucleotide associated at a faster rate and dissociated more slowly than the ΔUGT8RE1-50. Notably, after completing the entire set of injection cycles, the UGT8RE1-50 remained attached to the LHX6 at the sensor surface, while the ΔUGT8RE1-50 dissociated almost completely **(**Fig. 3E**).** When comparing steady-state binding, significantly stronger binding of the UGT8RE1-50 fragment was observed, with a steady-state dissociation constant of 2.57E-08 compared to 7.13E-08 for the ΔUGT8RE1-50 oligonucleotide **(**Fig. 3F**).** In this case, we discussed the sensorgrams primarily in qualitative terms, as the kinetic measurements and thus the quantitative analysis of the kinetics are challenging due to the unverified binding mechanism.

**Fig. 4:**
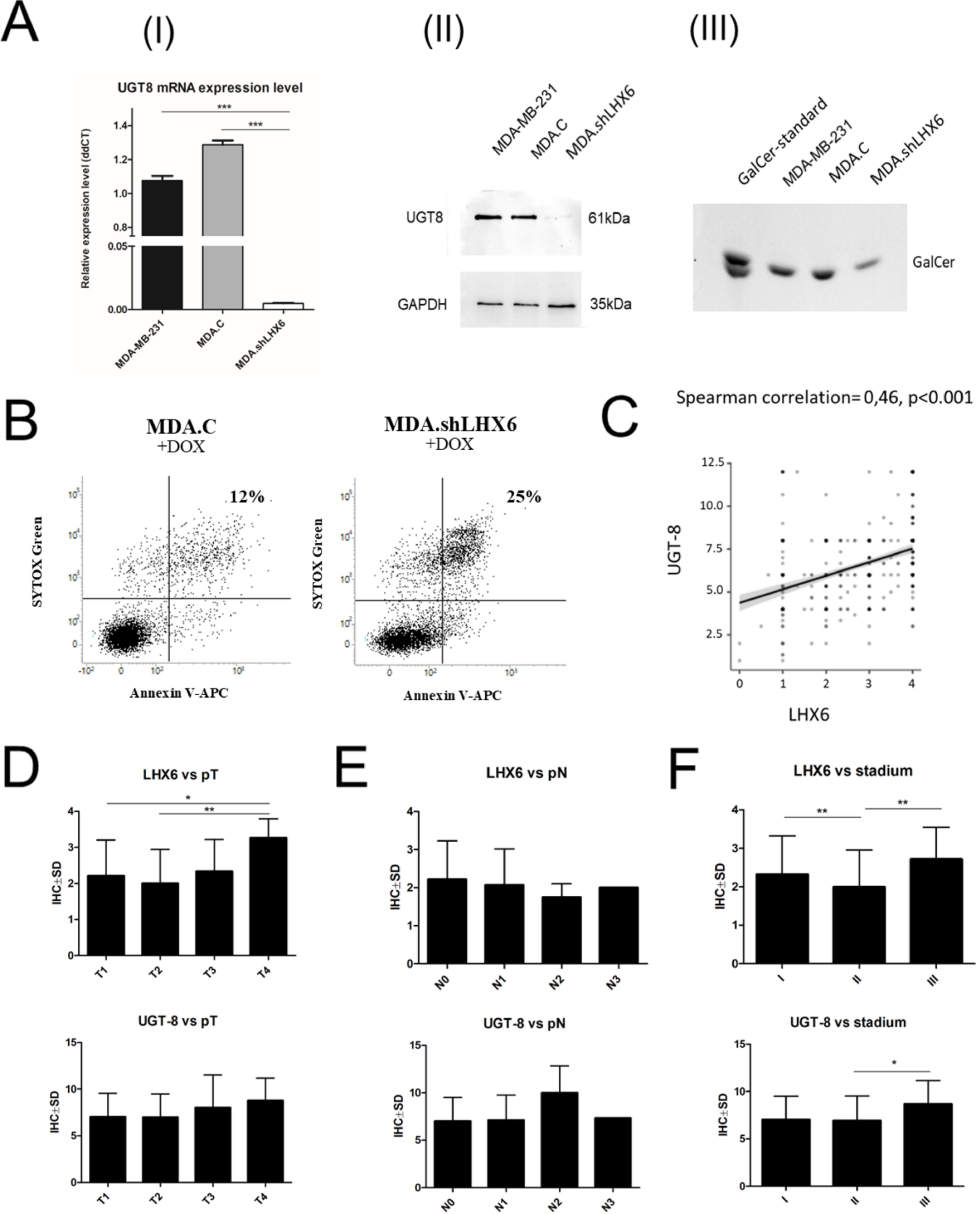
(**A**) Analysis of UGT8 mRNA expression levels (I), UGT8 protein levels (II), and GalCer levels (III) in wild-type MDA-MB-231 cells, MDA-MB-231 cells transfected with scrambled shRNA (MDA.C), and MDA-MB-231 cells transfected with shRNA directed against LHX6 mRNA (MDA.shLHX6). qPCR was used to analyze UGT8 mRNA. Its expression levels were normalized against GAPDH, and MDA.shLHX6 cells were used as a calibrator sample. All values are mean ± SD of at least 2 independent experiments, each assayed in triplicate. ***p < 0.001. UGT8 protein expression was analyzed by Western blotting. Rabbit polyclonal antibodies directed against UGT8 were used to detect UGT8. GalCer was analyzed by immunostaining of neutral GSLs separated by HP-TLC, with rabbit polyclonal anti-GalCer antibodies. For immunostaining, aliquots of total neutral GSLs corresponding to 1 × 10^7^ cells were applied to an HP-TLC plate. (**B**) Sensitivity of MDA-MB-231 cells treated with shRNA directed against LHX6 mRNA to apoptosis induced by doxorubicin. Cells were grown in the presence of doxorubicin at concentration of 0.5 μM for 48 h. Cellular response measured by staining with Annexin V and SYTOX Green. Flow cytometry dot plots show percentage of early apoptotic cells (Annexin V + /SYTOX Green − , lower right) and late apoptotic cells (Annexin V + /SYTOX Green + , upper right). Dot blots display representative results obtained from two independent experiments. (**C**) Positive correlation between UGT8 and LHX6 protein expression levels in invasive ductal carcinoma (IDC) (immunohistochemistry, IHC). r = 0.46, *p* < 0.001 (Spearman’s correlation). (**D**) IHC LHX6 and UGT8 expression in IDC tissues with regard to pT, * *p* < 0.05; ** *p* < 0.01. (**E**) IHC LHX6 and UGT8 expression in IDC tissues with regard to pN. (**F**) IHC LHX6 and UGT8 expression in IDC tissues with regard to clinical stadium, *p < 0.05; **p < 0.01.

To determine whether changes in *UGT8* gene expression are directly related to LHX6 levels, UGT8 expression was analyzed at the protein level in MDA-MB-231 cells treated with LHX6-specific shRNA. As expected, inhibition of LHX6 expression led to a significant reduction in UGT8 mRNA (Fig. 4A-I) as well as UGT8 protein levels (Fig. 4A-II). Notably, downregulation of UGT8 expression resulted in decreased GalCer levels in MDA-MB-231 cells (Fig. 4A-III). To assess the relationship between LHX6 expression and drug resistance in GalCer-positive BC cells, control MDA-MB-231 cells (MDA.C) and MDA-MB-231 cells treated with shRNAs targeting LHX6 mRNA (MDA.shLHX6) were incubated with doxorubicin and subjected to an apoptosis assay. Using APC–Annexin V and SYTOX Green staining, we found that the percentage of apoptotic cells after doxorubicin treatment was approximately two-fold lower (12%) in MDA.C cells, which express high levels of LHX6 and UGT8, compared with MDA.shLHX6 cells exhibiting low LHX6 and UGT8 expression (25%) (Fig. 4B).

### The expression of LHX6 correlates with the expression of UGT8 in BC tissue specimens

The study using cellular models was consistent with data obtained from immunohistochemical (IHC) analysis of LHX6 and UGT8 expression levels in invasive ductal carcinoma (IDC) tissue specimens. As expected, IHC staining showed nuclear localization of LHX6 and cytoplasmic localization of UGT8 (Figure S2A). Importantly, Spearman’s correlation analysis revealed a positive correlation between LHX6 and UGT8 protein expression (r = 0.46, *p* < 0.001) (Fig. 4C). Furthermore, in the analyzed BC cases, the expression levels of LHX6 and UGT8 were correlated with disease stage. More specifically, their expression levels correlated with pT, but not with pN (Fig. 4D,E). Information regarding pM was not available. LHX6 and UGT8 expression was also associated with the clinical stage of BC (Fig. 4F).

Based on literature indicating that UGT8 overexpression is significantly associated with a basal molecular signature in breast carcinomas^15^, which predominantly consist of triple-negative tumors^32^, we analyzed the correlation between UGT8 and LHX6 mRNA expression using publicly available datasets that contain molecular subtype classification. This analysis supported our IHC findings, as a positive correlation between UGT8 mRNA and LHX6 mRNA levels was observed in the basal molecular subtype (r = 0.2739, *p* < 0.0001; Spearman’s correlation; The Cancer Genome Atlas (TCGA), accessed via cBioPortal for Cancer Genomics) (Figure S2B). The expression levels of UGT8 and LHX6 mRNAs were also evaluated in the context of clinical outcomes. Patients with high UGT8 and LHX6 mRNA expression had significantly shorter overall survival (OS) and recurrence-free survival (RFS) compared with those showing low expression levels of both genes, respectively (Figure S2C and D).

## Discussion

Available data suggest that UGT8 expression is regulated at the transcriptional level in a cell- and tissue-specific manner^23–25^. Our results showed that in BC cells, UGT8 overexpression is also regulated at the transcriptional level and that the nucleotide sequences of the *UGT8* promoter region from human oligodendroglioma cells^22^ and human BC cells are the same. By comparing *UGT8* promoter activities between MDA-MB-231 cells, classified as 'mesenchymal-like’, and T47D and MCF7 cells, described as 'luminal epithelial-like’^33^, it was also confirmed that the *UGT8* gene promoter functions in a cell-specific manner^22^. In full agreement with previous studies on the expression of UGT8 at the mRNA and protein level in these cell lines^7^, the *UGT8* promoter activity in MDA-MB-231 cells was several times higher compared to T47D and MCF7 cells. The analysis of promoter activity using the series of deletion mutants allowed us to identify the functional region between -1132 to -1665 bp from the transcription start site, named UGT8 response element (UGT8RE), which contains strong enhancers required for overexpression of the *UGT8* gene in MDA-MB-231 cells. The search for TFs that bind to UGT8RE and play a key role in the up-regulation of UGT8 expression revealed highly increased expression of *LHX6* and *GSX1* genes at mRNA level in MDA-MB-231 cells compared to both T47D and MCF7 cells, but only in the case of *LHX6*, this was confirmed at the protein level. MDA-MB-231 cells also showed a strongly reduced expression of the SOX4 gene, which was reflected by the absence of SOX4 protein in the cell lysate, compared to T47D and MCF7 cells. Considering the lack of differences in GSX1 protein levels and, in the case of SOX4, EMSA results indicating binding of nuclear extracts from MDA-MB-231 cells to UGT8RE and thus the presence, not the absence, of specific TFs in the nuclear lysate, LHX6 was identified as the key regulatory protein responsible for the overexpression of UGT8 in BC cells representing a malignant "mesenchymal-like" phenotype^7^. This finding was supported by experiments using prUGT8 mutants with deletions of LHX6 binding sites. First, deletion of a single LHX6 binding site, designated LHX6BS2, markedly reduced prUGT8 activity. Second, EMSA experiments revealed that shRNA-mediated inhibition of LHX6 expression prevented binding of nuclear extracts to the UGT8RE3 fragment of prUGT8. Third, SPR analysis showed that a recombinant human LHX6 protein corresponding to amino acids 274–363 bound to a prUGT8 fragment containing an intact LHX6 binding site, in contrast to its deletion mutant. However, it should be noted that direct in vivo binding of LHX6 to the UGT8 regulatory region has not been demonstrated by chromatin immunoprecipitation (ChIP), which represents a limitation of this study. The involvement of LHX6 in the up-regulation of UGT8 expression in BC cells was further supported by functional studies. Inhibition of LHX6 expression by shRNA decreased UGT8 expression, resulting in reduced synthesis of GalCer. Consistent with previous reports^18,20^, reduced GalCer levels increased the sensitivity of BC cells to doxorubicin-induced apoptosis.

The *LHX6* gene encodes a LIM-homeodomain protein characterized by the presence of two LIM domains (cysteine-rich motifs involved in intra- and intermolecular interactions) in addition to the homeodomain^34^. Originally, LHX6 protein was shown to control the patterning and differentiation of orofacial structures and to be involved in the development of the forebrain of mammalian embryos. The LHX6 gene is also expressed in various normal adult tissues, and its mRNA presence in tumor tissues was first described in head and neck and colorectal cancer^35^. Subsequent studies revealed that the *LHX6* gene was downregulated in lung and breast cancers compared to normal corresponding tissues^36–38^. Using cellular models, this TF was found to inhibit cell proliferation, colony formation, and migration in vitro and suppress tumorigenicity and metastatic potential in vivo. Based on this data, it was proposed that LHX6 acts as a tumor suppressor gene. This is in contrast to our results, as high expression of LHX6 was functionally related to increased viability and drug resistance of BC cells. These discrepancies can be linked to different characteristics of BC cell lines used, as Hu and Xie^38^ and Bi et al.^39^, in contrast to us (see above), did not find differences in LHX6 expression between 'mesenchymal-like’ MDA-MB-231 cells and 'luminal epithelial-like’ T47D and MCF7 cells. This different characteristic is also reflected by the highly invasive phenotype of T47D and MCF7 cells used by them, which are generally described as low or non-invasive^40–42^. According to the authors^11,38^, at the molecular level, LHX6 suppresses tumor progression through transcriptional inhibition of β-catenin expression, which results in suppression of Wnt/β-catenin signaling pathway. It was also suggested that overexpression of LHX6 could inhibit the activation of PI3K/AKT/mTOR signaling pathway^39^. However, it should be emphasized that the direct role of LHX6 in the regulation of the *CTNNB1* gene, as well as the regulation of PI3K/AKT/mTOR signaling, has not been carefully evaluated. In NSCLC, the key TF driving UGT8 expression was found to be SOX9^25^. However, these differences between BC and NSCLC cells are not surprising, since in the latter UGT8 plays a role as a positive regulator of proliferation, also affecting their migration and invasiveness, and importantly, did not affect the apoptotic properties of NSCLC cells, which is in opposite to BC cells. Therefore, these contrasting results once again highlight the cell-specific regulation of the *UGT8* gene promoter and link it to specific biological properties of cells.

It was found previously that UGT8 is overexpressed in a subset of tumors representing the basal-like molecular subtype and associated with an increased risk of lung metastasis^7,16,17^. Therefore, the expression levels of LHX6 and UGT8 were analyzed in IDC tissue specimens, although primary tumors with matched lung metastases were not available in this study. Generally, the IHC analysis of LHX6 and UGT8 expression levels in IDC tissue specimens confirmed data obtained—using cellular models, showing the positive correlation between LHX6 and UGT8 expression levels.

## Conclusions

Our data showed that overexpression of UGT8 and accumulation of GalCer, leading to drug resistance of BC, is the result of differences in the expression of the LHX6 homeobox protein. Importantly, we have shown previously that overexpression of UGT8 is associated with multidrug resistance of BC cells and that GalCer acts as an anti-apoptotic molecule^20^. For these reasons, understanding the molecular mechanisms involved in the regulation of *UGT8* gene expression, which are responsible for the highly increased expression of UGT8 in a subset of BC tumors with increased risk of lung metastasis, is of great importance not only to gain insight into the pathological conditions, but also to develop new therapeutic strategies.

## Supplementary Information

Below is the link to the electronic supplementary material.

Supplementary Material 1

Supplementary Material 2

Supplementary Material 3

Supplementary Material 4

Supplementary Material 5

Supplementary Material 6

Supplementary Material 7

Supplementary Material 8

## Data Availability

The datasets supporting the findings of this study are available in the Repository of Wrocław University of Environmental and Life Sciences (10.57755/g512-ze17). The UGT8 promoter sequence is available in GenBank (accession no. PZ019607). All supporting data and protocols have been provided within the article or through supplementary data files. Further datasets used and/or analysed during the current study are available from the corresponding author on reasonable request.

## Abbreviations

ATCC: American Type Culture Collection
BC: Breast cancer
EMSA: Electrophoretic mobility shift assay
GalCer: Galactosyloceramide
GSL: Glycosphingolipid
IDC: Invasive ductal carcinoma
IHC: Immunohistochemistry
IRS: Semiquantitative immunoreactive score
LHX6BS: LHX6 binding site
NSCLC: Non-small lung cancer
SPR: Surface plasmon resonance
TF: Transcription factor
TLC: Thin-layer chromatography
TNBC: Triple-negative breast cancer
UGT8: Ceramide galactosyltransferase
UGT8RE: UGT8 response element
WHO: World Health Organization
