# Supplementary figures and images for: UGT8/GalCer-dependent resistance of breast cancer cells to drug-induced apoptosis is potentially regulated by the LIM/homeobox protein LHX6
Source: Sci Rep. 2026 Mar 4;16:11934. doi: 10.1038/s41598-026-42260-1 (PMC13068971; doi:10.1038/s41598-026-42260-1)

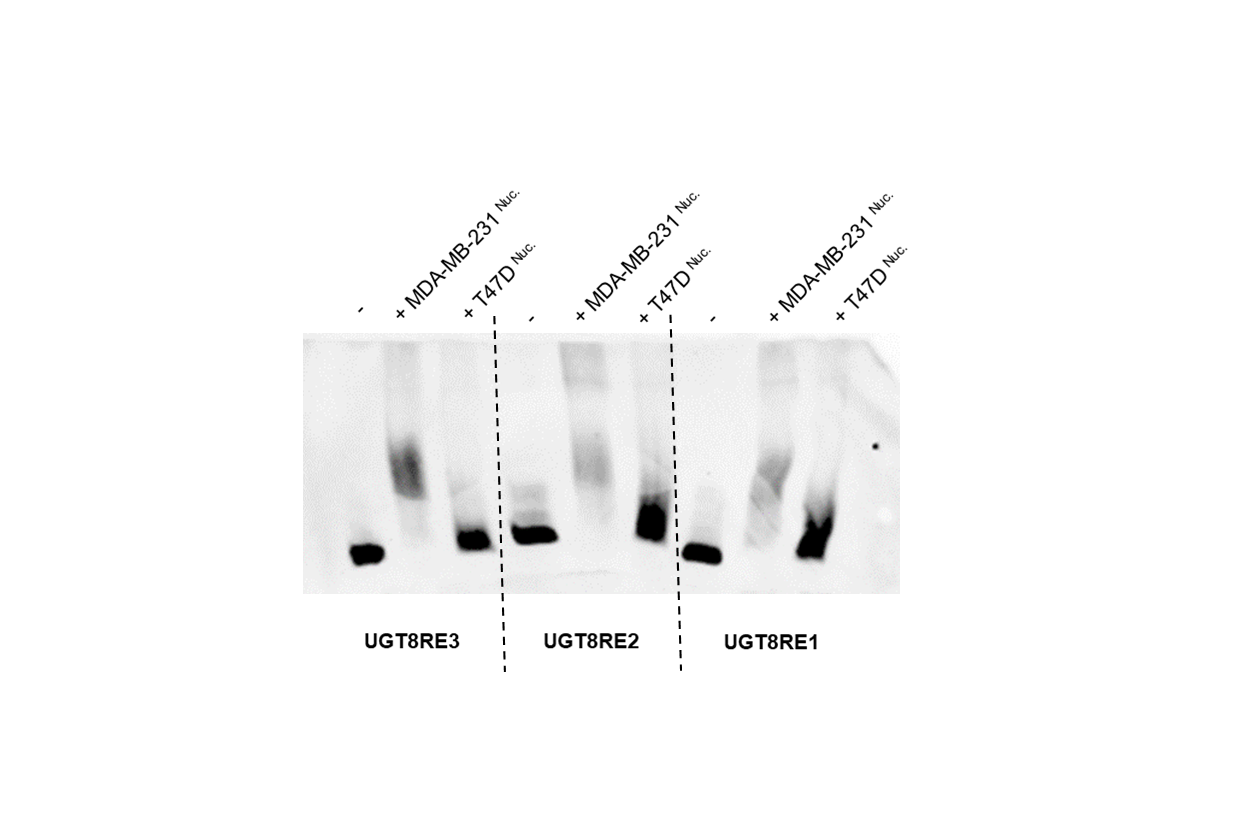

Supplement: Supplementary file 1 — Supplementary Material 1 [file 41598_2026_42260_MOESM1_ESM.tif]

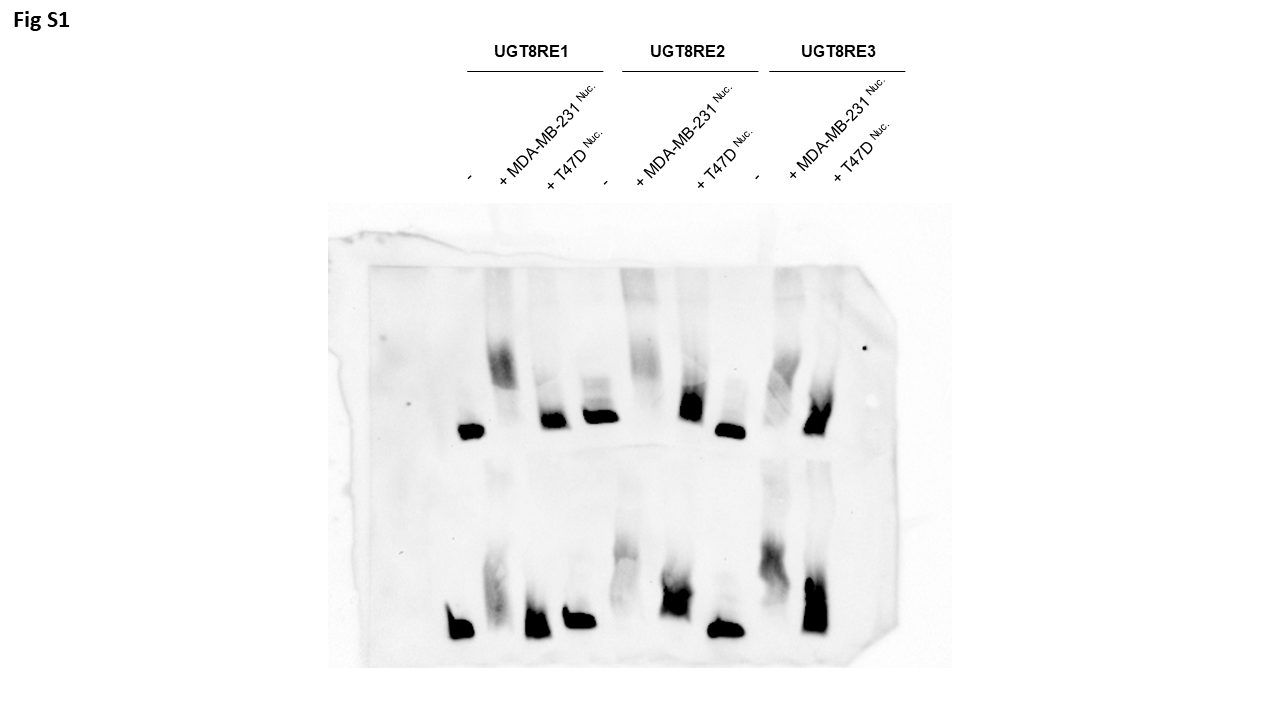

Supplement: Supplementary file 3 — Supplementary Material 3 [file 41598_2026_42260_MOESM3_ESM.tif]

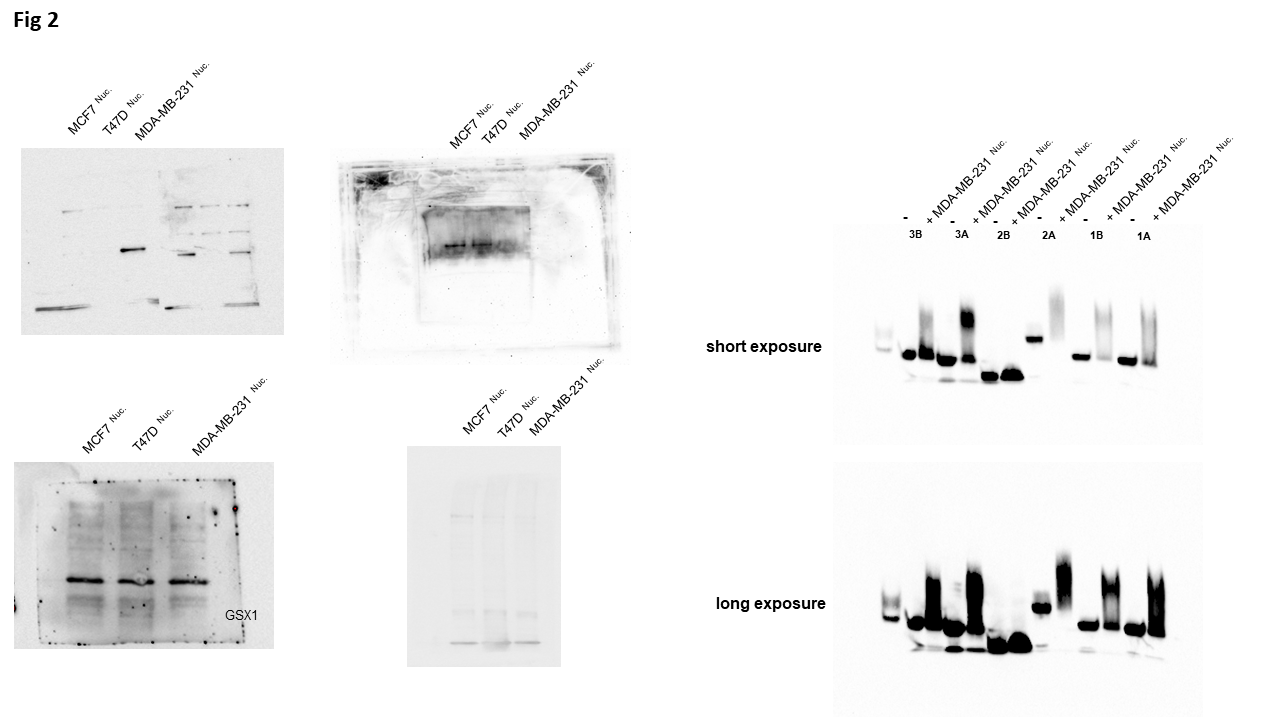

Supplement: Supplementary file 4 — Supplementary Material 4 [file 41598_2026_42260_MOESM4_ESM.tif]

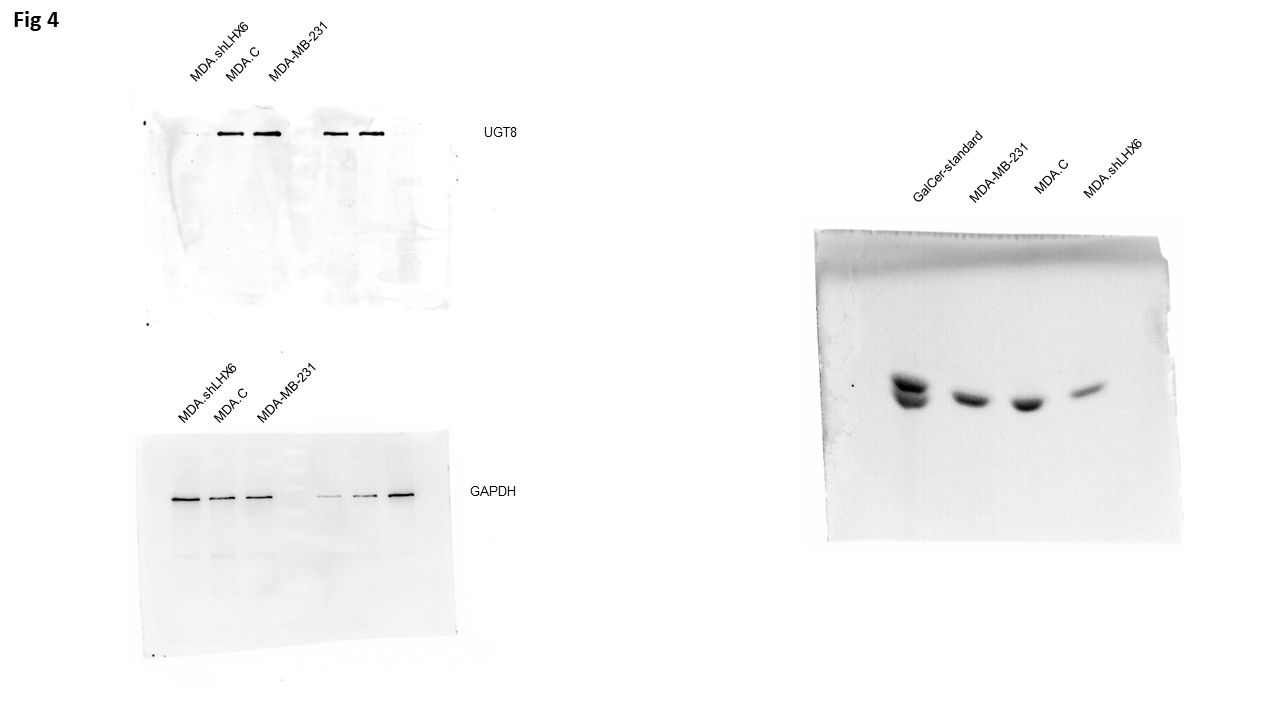

Supplement: Supplementary file 5 — Supplementary Material 5 [file 41598_2026_42260_MOESM5_ESM.tif]

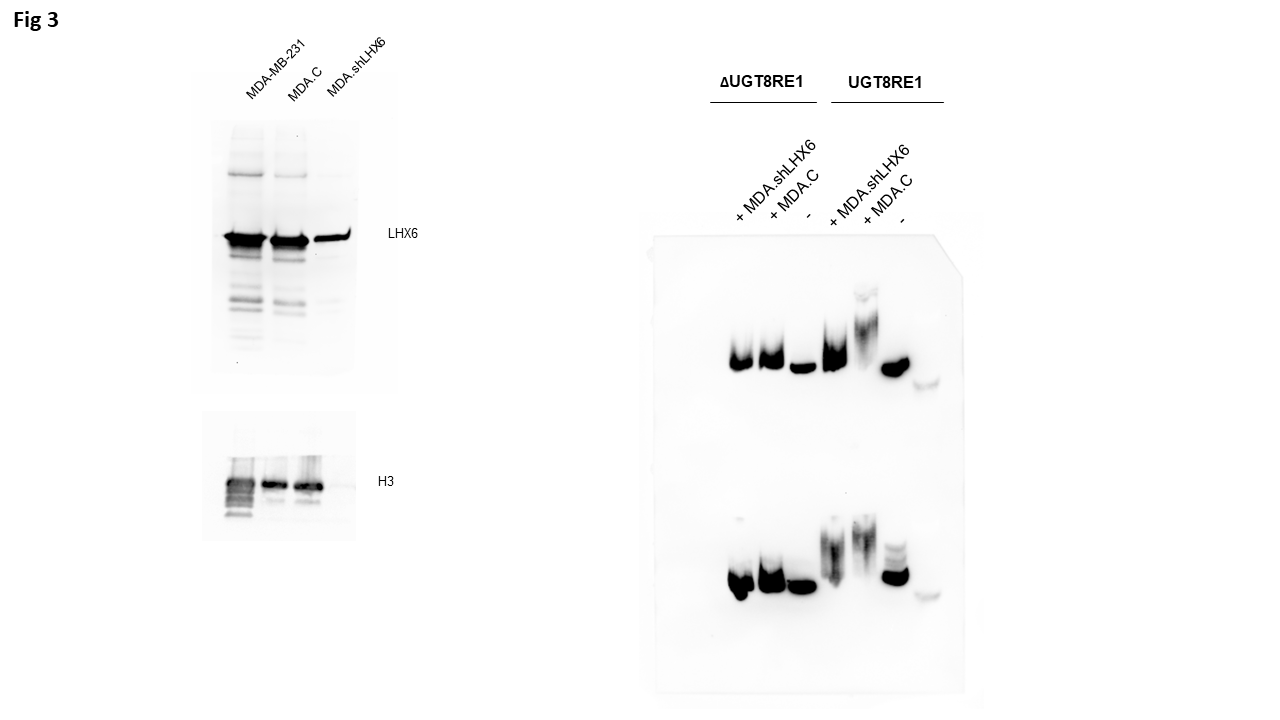

Supplement: Supplementary file 6 — Supplementary Material 6 [file 41598_2026_42260_MOESM6_ESM.tif]

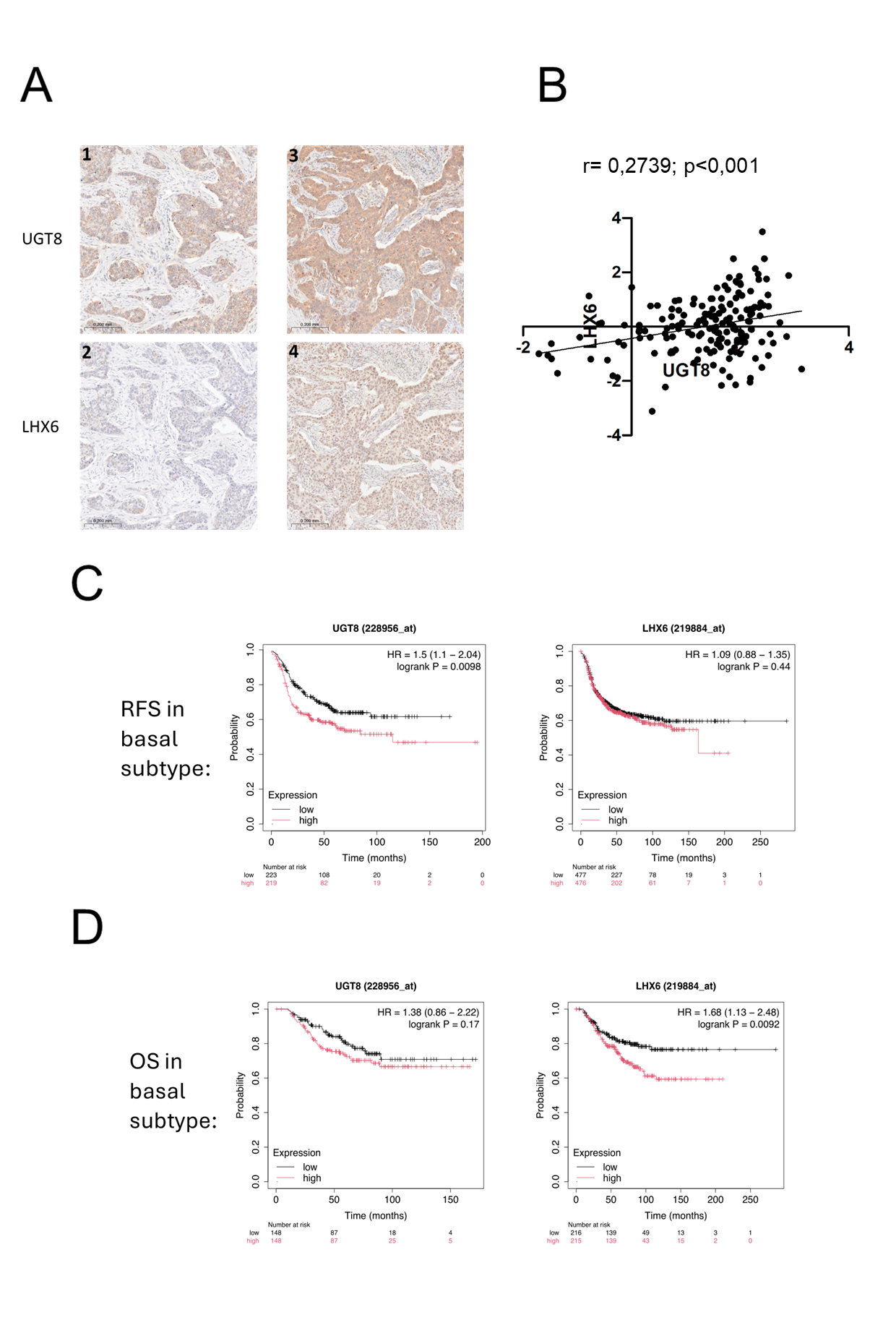

Supplement: Supplementary file 7 — Supplementary Material 7 [file 41598_2026_42260_MOESM7_ESM.tif]
