# Supplementary material for: UGT8/GalCer-dependent resistance of breast cancer cells to drug-induced apoptosis is potentially regulated by the LIM/homeobox protein LHX6
Source: Sci Rep. 2026 Mar 4;16:11934. doi: 10.1038/s41598-026-42260-1 (PMC13068971; doi:10.1038/s41598-026-42260-1)
Supplement: Supplementary file 2 — Supplementary Material 2 [file 41598_2026_42260_MOESM2_ESM.docx]

**Electrophoretic mobility shift assay (EMSA)**

| **UGT8 response element** | **5’ biotin-labeled primer** | **primer sequence 5’-3’** | **primer length** |
| --- | --- | --- | --- |
| UGT8RE1A | forUGT8RE1A | [Btn]AACACCCTTACTGTGTAGAGCTG | **23** |
|  | revUGT8RE1A | [Btn]GTATTCTCAATTGTTATTTTTTTCAGAAATACT | **33** |
| UGT8RE1B | forUGT8RE1B | [Btn]TGATATATATAGTCCACAGAAATAATGAGGGA | **32** |
|  | revUGT8RE1B | [Btn]GGTGTAAACTGACCATTGTGTAAGG | **25** |
| UGT8RE2A | forUGT8RE2A | [Btn]CCTTACACAATGGTCAGTTTACACC | **25** |
|  | revUGT8RE2A | [Btn]TGTTTAAAATTGTTTCACCACATCCTTG | **28** |
| UGT8RE2B | forUGT8RE2B | [Btn]TTTTTATATAATAAAAGAAAAAAGTTGTTTTAATTG | **36** |
|  | revUGT8RE2B | [Btn]GGTCAAGCTTCAGTTTCAAGCT | **22** |
| UGT8RE3A | forUGT8RE3A | [Btn]AGCTTGAAACTGAAGCTTGACC | **22** |
|  | revUGT8RE3A | [Btn]GTAACATAATAAAGAAAAGAGAATTTAGAC | **30** |
| UGT8RE3B | forUGT8RE3B | [Btn]ATTTCATTTACTTTAGGTTTTTAGTGC | **27** |
|  | revUGT8RE3B | [Btn]AGCCTTTGTACCTGATTACCATACT | **25** |

**Promotor insertion into pGL3-Basic luciferase vector**

| **primer** | **primer sequence 5’-3’** | **primer length** |
| --- | --- | --- |
| forMluI-prUGT8 | CA**ACGCGT**CACATATGGTTGTTTATATTTTCCA | **33** |
| revNheI-prUGT8 | GA**GCTAGC**TTTATCATCAAGGCGGCTC  **Mlu I** | **27** |
| forMluI-prUGT8(-341) | TTT**ACGCGT**TCCGCTACAAGAGAGCCAGA  **Nhe I** | **29** |
| forMluI-prUGT8(-542) | TTT**ACGCGT**GTTGCTTGCTGGTCAAGACG  **Mlu I** | **29** |
| forMluI-prUGT8(-763) | TTT**ACGCGT**TCTGGAATTTATGCTCGACACGA  **Mlu I** | **32** |
| forMluI-prUGT8(-1008) | TTT**ACGCGT**CCATCCATGAACATTCTTGAGCA  **Mlu I** | **32** |
| forMluI-prUGT8(-1132) | TTT**ACGCGT**TGGTAATCAGGTACAAAGGCTGT  **Mlu I** | **32** |
| forMluI-prUGT8(-1240) | TTT**ACGCGT**AGGACAAGGATGTGGTGAAACA  **Mlu I** | **31** |
| forMluI-prUGT8(-1665) | TTT**ACGCGT**GGGCGGTGAAATGAACCTCA  **Mlu I** | **29** |
| forMluI-prUGT8(-1858) | TTT**ACGCGT**ATCTGAATGGGAGCTTGAAGGA  **Mlu I** | **31** |

**Mlu I**

Highlighted is the DNA sequence recognized by Mlu I restriction enzyme

**Real-time-PCR assay**

| **gene** | **primer** | **primer sequence 5’-3’** | **product length** |
| --- | --- | --- | --- |
| SOX4 | SOX4-F | GACCTGAACCCCAGCTCAAA | 100 |
|  | SOX4-R | AGCCGGGCTCGAAGTTAAAA |  |
| GSX1 | GSX1-F | CGCTCTACCAGACCTCCTAC | 172 |
|  | GSX1-R | AGGCGGGACAGGTACATATT |  |
| EVX1 | EVX1-F | CCGAGTACCAGCACAGCAAAG | 178 |
|  | EVX1-R | ATCTGCTCTCGGGTGAAGGC |  |
| LHX6 | LHX6-F | TGCGGCCTCGAGATCCT | 180 |
|  | LHX6-R | GGCACACTTGGTCCCGAAT |  |
| NKX6-3 | NKX6-3-F | GCTCGCAGGGGGTCTACTA | 178 |
|  | NKX6-3-R | CGGGTGTGCTTCTTCTTGTG |  |
| MEOX1 | MEOX1-F | GGCGGAGAAAGGAGAGTTCA | 199 |
|  | MEOX1-R | TTGACCTGGCGCTCAGAGA |  |
| MEOX2 | MEOX2-F | CGGCAAGAGGAAAAGCGACA | 75 |
|  | MEOX2-R | TTCCTGGGTTTGCTGTTGACT |  |
| POU5F1B | POU5F1B-F | ATGCAGGCCCGAAAGAGAAA | 102 |
|  | POU5F1B-R | GCTGATCTGCAGTGTGGGTT |  |
| POU3F4 | POU3F4-F | ACCATTGCCAGGATCACTCC | 141 |
|  | POU3F4-R | TACCATACAGTGTGCCCAGC |  |
| UGT8 | UGT8-F | CATGGTGTGCCTGTAGTGG | 172 |
|  | UGT8-R | GAGCCCTCTGACGGTAGC |  |
| GAPDH | GAPDH-F | GGAAGGTGAAGGTCGGAGTC | 108 |
|  | GAPDH-R | TGAAGGGGTCATTGATGGCA |  |

**DNA hybridization for SPR assay**

| **DNA fragment** | **Probe** | **single-stranded DNA sequence 5’-3’** | **primer length** |
| --- | --- | --- | --- |
| ΔUGT8RE1-50 | sense | AAGAAATAAATAGAGAGTAT**GGTAATCAGG**TACAAAGGCTGTGAAAATAT | 50 |
|  | antisense | ATATTTTCACAGCCTTTGTACCTGATTACCATACTCTCTATTTATTTCTT | 50 |
| UGT8RE1-50 | sense | AAGAAATAAATAGAGAGTATTACAAAGGCTGTGAAAATATTCCCTGCATT | 50 |
|  | antisense | AATGCAGGGAATATTTTCACAGCCTTTGTAATACTCTCTATTTATTTCTT | 50 |

Highlighted is the DNA sequence recognized by LHX6.
