# Supplementary material for: UGT8/GalCer-dependent resistance of breast cancer cells to drug-induced apoptosis is potentially regulated by the LIM/homeobox protein LHX6
Source: Sci Rep. 2026 Mar 4;16:11934. doi: 10.1038/s41598-026-42260-1 (PMC13068971; doi:10.1038/s41598-026-42260-1)
Supplement: Supplementary file 8 — Supplementary Material 8 [file 41598_2026_42260_MOESM8_ESM.docx]

**Supplementary Materials for Suchanski et al.**

**UGT8/GalCer-Dependent Resistance of Breast Cancer Cells to Drug-Induced Apoptosis is Regulated by the LIM/Homeobox Protein LHX6**

**Summary of Supplementary Materials:**

**Supplementary Figures**

**Figure S1**. EMSA results showing the binding of nuclear extracts from MDA-MB-231 cells to three DNA fragments corresponding to nucleotides −1618 to −1451 (UGT8RE1), −1450 to −1281 (UGT8RE2), and −1280 to −1124 (UGT8RE3).

**Figure S2.** (**A**) Immunohistochemistry (IHC) staining demonstrates nuclear localization of LHX6 and cytoplasmic localization of UGT8 in representative cases of breast carcinoma. (**B**) A positive correlation between UGT8 mRNA and LHX6 mRNA levels in the basal molecular subtype of breast carcinoma (**C, D**) Correlation of UGT8 and LHX6 expression with survival outcomes in basal molecular subtype of breast carcinoma.

**Supplementary Tables**

Table S1: List of primers used in this study

**Figure legends**

**Figure S1**. Binding of nuclear proteins from MDA-MB-231 and T47D cells to UGT8 gene promoter regions designated as UGT8 response elements (UGT8RE): UGT8RE1, UGT8RE2, and UGT8RE3, analyzed by EMSA in the presence (+) or absence (–) of nuclear extracts. EMSA was performed using double-stranded, biotin-labeled oligonucleotide probes.

**Figure S2.** **(A)** Immunohistochemical localization of UGT8 (cytoplasm) and LHX6 (nucleus) in serial sections of breast carcinomas (×100 magnification). Panels 1 and 2: weak UGT8 staining correlates with weak LHX6 staining. Panels 3 and 4: strong UGT8 staining correlates with strong LHX6 staining. **(B)** Positive correlation between UGT8 mRNA and LHX6 mRNA levels (r = 0.2739, p < 0.0001; Spearman’s correlation) in the basal molecular subtype of breast carcinoma (n = 171) from the TCGA BRCA cohort (n = 1082; TCGA Consortium, Nature 2012, 490:61–70). **(C)** Univariate Kaplan–Meier analysis of recurrence-free survival (RFS) and **(D)** overall survival (OS) in patients representing the basal molecular subtype of breast carcinoma, as identified by the PAM50 molecular signature (n = 442), from the “KMplotter” multi-study breast cancer cohort (https://kmplot.com; Posta M, Gyorffy B, Br. J. Pharmacol. 2025).
